# The association between serum uric acid and hypertriglyceridemia: evidence from the national health and nutrition examination survey (2007–2018)

**DOI:** 10.3389/fendo.2023.1215521

**Published:** 2023-07-18

**Authors:** Mo-Yao Tan, Chao-Yue Mo, Fang Li, Qian Zhao

**Affiliations:** ^1^ Chengdu Integrated TCM and Western Medicine Hospital, Chengdu, China; ^2^ College of Life and Science, Chengdu University of Traditional Chinese Medicine, Chengdu, Sichuan, China; ^3^ Clinical Medical School, Chengdu University of Traditional Chinese Medicine, Chengdu, Sichuan, China

**Keywords:** serum uric acid, hypertriglyceridemia, NHANES, weighted logistic regression analysis, subgroup analysis

## Abstract

**Background:**

Accumulating evidence suggests that elevated serum uric acid (SUA) may be a risk factor for hypertriglyceridemia (HTG). However, the epidemiological evidence for the association between SUA and HTG is limited. This article aimed to use the data from National Health and Nutrition Examination Survey (NHANES) (2007–2018) database to bridge the research gap.

**Methods:**

This cross-sectional study used data from 10027 adults involved in NHANES from 2007-2018. We designed the exposure variable as SUA and the outcome variable as HTG. The covariates included demographics, questionnaires, laboratory, and examination information. Weighted logistic regression and subgroup analysis were used to explore the independent association between SUA and HTG. Furthermore, interaction tests were also carried out to evaluate the strata differences. Generalized additive models (GAM), smooth curve fittings, and threshold effect analysis were applied to examine the non-linear relationship.

**Results:**

A total of 10027 participants were included, of which 3864 were HTG participants and 6163 were non-HTG participants. After fully adjusting for confounders, weighted multiple logistic regression models revealed a 77% increase in the risk of HTG when each unit of log2-SUA increased. There was also a positive association between elevated log2-SUA and developed risk of HTG in the quartile (Q) groups (Q1 OR: 1.00; Q2 OR: 1.17 [95%CI: 0.95,1.45]; Q3 OR: 1.43 [95%CI: 1.16,1.78]; Q4 OR: 1.68 [95%CI: 1.36,2.08]. The subgroup analysis results remained consistent across strata, with a strong positive correlation between SUA and HTG. Interaction tests showed no dependence on physical activity (PA), gender, BMI, smoking status, alcohol intake, hypertension, and diabetes for this positive association between log2-SUA and HTG (all *p* for interaction >0.05). The participants’ age may impact the strength of the association between SUA and HTG (*p* for interaction <0.05).

**Conclusion:**

There is a positive association between SUA and HTG in US adults. Considering that SUA may be a risk factor for HTG, individuals diagnosed with HTG should prioritize the daily management of SUA as part of their comprehensive care.

## Introduction

Hypertriglyceridemia (HTG), characterized by fasting serum triglyceride (TG) levels exceeding 1.7 mmol/L (1), represents a prevalent lipid metabolism disorder (2). The development of HTG involves a complex interplay of genetic and non-genetic factors (3), leading to its classification into primary HTG and secondary HTG based on the underlying etiology (4). According to NHANES data, it is estimated that there are approximately 10.84 million individuals diagnosed with HTG in the United States (5). Moreover, severe HTG has been linked to a substantial rise in healthcare costs, ranging from 33% to 38% annually (6), thereby significantly amplifying the societal healthcare burden. Studies have shown that HTG poses not only a risk for pancreatitis and cardiovascular disease but also exhibits a robust association with obesity, diabetes, and nonalcoholic fatty liver disease (NAFLD) (7–9). Notable is the potential link between NAFLD and bladder cancer development (10). In a comprehensive cohort study, researchers observed that elevated serum triglyceride (TG) levels were independently associated with a more severe progression of pancreatitis and a higher likelihood of complications (11). Furthermore, a previous study revealed that serum TG levels reaching 10.20 mmol/L or higher were linked to a 10% to 20% increased risk of pancreatitis (12). The implementation of lifestyle interventions, such as dietary modifications, weight management, and increased physical activity, is considered the primary and most valuable approach for treating HTG. By significantly lowering TG levels through these interventions, it is possible to not only prevent pancreatitis but also reduce the risk of cardiovascular disease (2, 13, 14).

SUA is the final oxidation product of exogenous and endogenous purine metabolism and is produced in the intestine, liver, and muscle (15–17). Endogenous purines are the main component of purines in the body, accounting for 80% of total purines, which are mainly derived from the oxidative breakdown of the body’s nucleic acids (16). Exogenous purines are derived from dietary intake, including seafood, fatty and organ meats (e.g., liver and kidney), fructose, and alcohol (18). In recent years, there has been an observed increase in people’s SUA levels, leading to the emergence of hyperuricemia as a significant public health concern. Previous data have indicated that the prevalence of hyperuricemia in the United States is as high as approximately 20% (19). Notably, research has demonstrated an association between hyperuricemia and urological cancer (20). This may be related to dietary habits, lifestyle changes, and medication use (21, 22). Over the years, numerous studies have consistently demonstrated a strong association between high SUA levels and the development of cardiovascular disease. Furthermore, elevated SUA has been linked to increased risks of all-cause mortality and cardiovascular mortality (23, 24). High SUA is also associated with the development of many other diseases, including diabetes, hypertension, NAFLD and kidney disease (25–27). Above all, elevated SUA levels can alter the body’s physiopathology and heighten the susceptibility to diseases.

One hypothesis is that SUA levels may be related to HTG. It is thought-provoking that both HTG and high levels of SUA can lead to the development of NAFLD (9, 27). Gout patients have been shown to have an increased risk of urologic cancers (20), and HTG-induced NAFLD has also been shown to indirectly raise this risk (10). Research investigating the relationship between SUA and metabolic syndrome (MetS) found that hyperuricemia showed the strongest association with high TG (PR = 2.32, 95% CI: 1.84-2.91) (28). A 5-year cohort study conducted in Japan revealed an increased risk of low-density lipoprotein (LDL) and HTG with elevated SUA levels (29). Additionally, previous evidence indicates that elevated SUA levels can induce mitochondrial abnormalities, contributing to the progression of HTG (8). Notably, an animal experiment demonstrated lower lipase activity in the high SUA group compared to the low SUA group (30). Decreased lipase activity is associated with reduced TG catabolism (31). Moreover, apolipoprotein E (ApoE) has been implicated in SUA-induced HTG (32).

Drawing from the existing evidence, it is suggested that SUA could serve as a risk factor for HTG. Accordingly, we collected NHANES data (2007–2018) to provide epidemiological evidence, and conducted an investigation into the relationship between SUA and HTG by employing weighted multivariate logistic regression and performing subgroup analysis.

## Materials and methods

### Data source

The NHANES database is a nationally conducted cross-sectional study aiming to evaluate the health and nutritional status of non-institutionalized residents in the United States. Administered by the National Center for Health Statistics (NCHS), NHANES collects data through interviews and examinations. The study design employs a stratified multistage probability sampling method, ensuring a highly representative sample. The NHANES protocol has been reviewed and approved by the Research Ethics Review Committee of the National Center for Health Statistics, and all participants have provided written informed consent. The publicly available data used in our analysis can be accessed at https://www.cdc.gov/nchs/nhanes/. This study adhered strictly to the Strengthening the Reporting of Observational Studies in Epidemiology (STROBE) principle for cross-sectional studies (**Supplementary material 1**) (33).

### Study population

**Figure 1:**
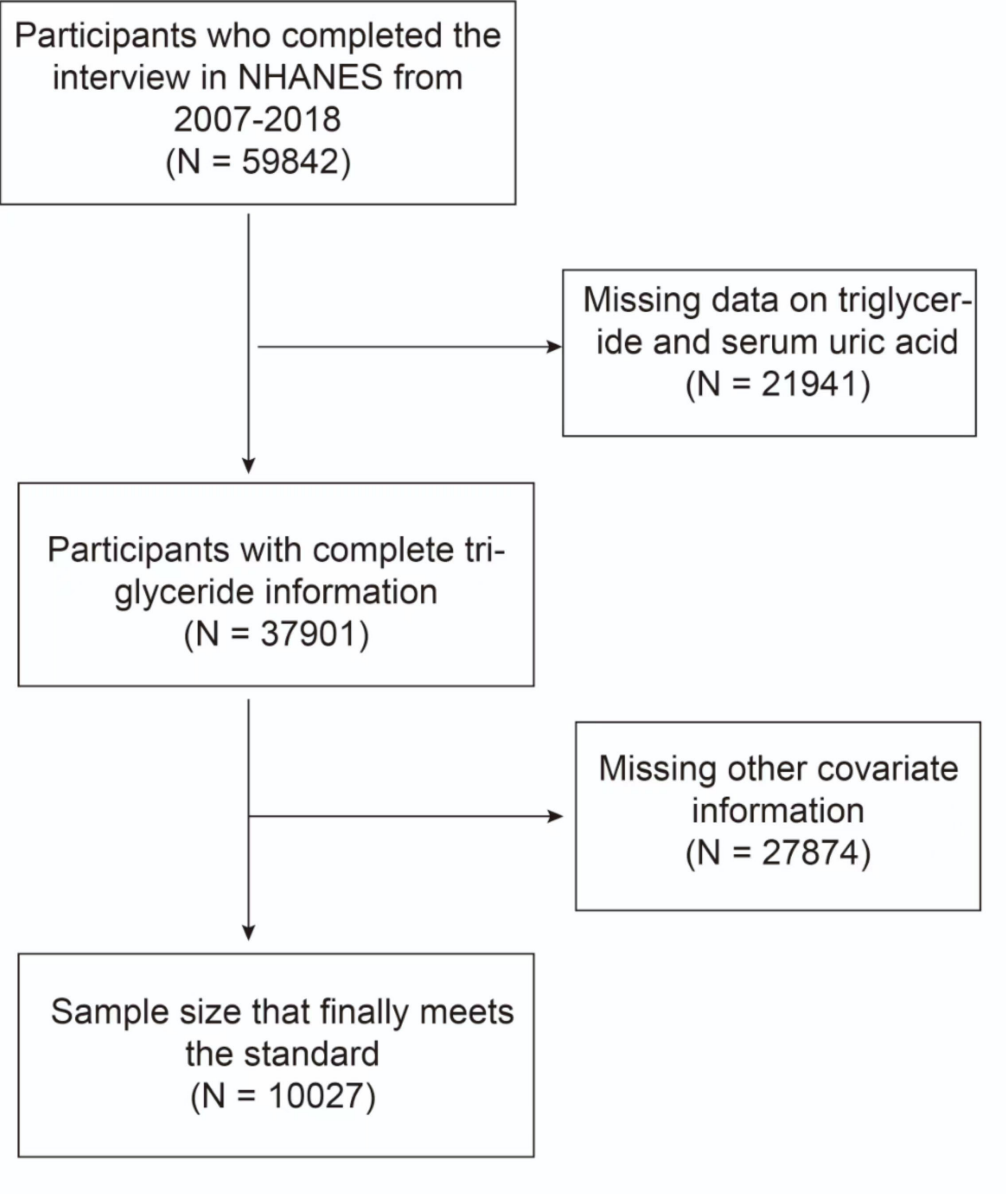
Flowchart of the sample selection from the 2007–2018 National Health and Nutrition Examination Survey (NHANES).

The study incorporated data from six cross-sectional cycles (2007–2018) of NHANES. The initial inclusion of 59842 participants in the study was based on the inclusion criteria that participants were at least 20 years old and that SUA and HTG data was available. To ensure data integrity, participants with missing data on prescription for cholesterol (PFC), alcohol intake, the ratio of family income to poverty (PIR), education level, body mass index (BMI), and sedentary time were excluded. Ultimately, a total of 10,027 participants who satisfied the aforementioned inclusion and exclusion criteria were included in the data analysis (**Figure 1**).

### Measurements and definition of variables

#### Exposure variable and outcome variable

SUA was used as the exposure variable in this study. To account for its right-skewed distribution, log2 transformation was applied to SUA during subgroup and regression analyses. HTG was defined as an outcome variable, with HTG being classified as serum TG content greater than or equal to 1.7 mmol/L, according to endocrine clinical guidelines (4).

#### Covariates

Based on prior research and clinical experience, we have incorporated the following summary of covariates that might influence the relationship between SUA and HTG (34, 35). The study considered the following continuous covariates: age (years), sedentary time (minutes), Alanine aminotransferase (ALT, U/L), Aspartate aminotransferase (AST, U/L), creatinine (µmol/L), blood urea nitrogen (mmol/L), and total cholesterol (mmol/L). Categorical variables included: gender (Male/Female), race (Mexican American/other Hispanic/Non-Hispanic White/Non-Hispanic Black/Other Race), educational level (High school or above high school/less than High school), smoking status (Yes: smoking at least 100 cigarettes; No: smoking less than 100 cigarettes), PA (yes was defined as engaging in any moderate recreational activities for at least 10 continuous minutes), hypertension (yes/no), diabetes (yes/no), prescription for cholesterol (PFC) (yes/no), and ratio of family income to poverty (PIR). Hypertension was defined as an average systolic blood pressure ≥130mmHg or diastolic blood pressure ≤ 80mmHg, or taking medication for hypertension (36). Diabetes was diagnosed based on three criteria (1): self-reported diagnosis by a physician or healthcare professional (2), HbA1c (glycated hemoglobin) level over 6.5%, and (3) fasting blood glucose (FPG) level over 126 mg/dL (37). PIR was classified as low-income (PIR ≤ 1.3), middle-income (PIR > 1.3–3.5), and high-income (PIR> 3.5) (38). Marital status was divided into living alone, married, or living with a partner (34). Alcohol intake was classified as mild, moderate, and heavy. Heavy alcohol use was defined as ≥3 drinks per day for females or ≥ four drinks per day for males (39). Moderate alcohol use was defined as 2-3 drinks per day for females and 3-4 drinks per day for males. Mild alcohol use was regarded as others (40). The BMI was categorized as underweight or normal (<25 kg/m^2^), overweight (≥25 to <30 kg/m^2^), and obese (≥30 kg/m^2^) (38).

### Statistical analysis

All statistical analyses were conducted according to CDC guidelines. Considering the complicated multistage cluster survey design, NHANES-generated sampling statistics strata, clusters, and weights were used to ensure the results were generalizable to the U.S. population (41).

Continuous variables were presented as mean with standard deviation (SD), while categorical variables were expressed as percentages. To address the right-skewed distribution of SUA data, log2 transformation was applied for regression and subgroup analysis. The statistical analysis comprised four main steps, aiming to examine the association between SUA levels and HTG among the selected participants. Firstly, participants’ TG levels were categorized into HTG and non-HTG groups based on clinical guidelines. Differences between these groups were assessed using the chi-square test for categorical variables and the weighted Student’s t-test for continuous variables. In the second step, weighted multivariate logistic regression models were employed to examine the independent association between SUA and HTG in three models. Model 1 did not include any covariate adjustments. Model 2 was adjusted for gender, age, and race. Model 3 included adjustments for all covariates, including age, gender, race, education level, sedentary time, AST, ALT, creatinine, blood urea nitrogen, total cholesterol, PIR, body mass index (BMI), smoking status, alcohol intake, physical activity (PA), hypertension, diabetes, and PFC. Furthermore, SUA was transformed from a continuous variable to a categorical variable (Q) for further analysis. In the third step, a subgroup analysis was conducted to examine the impact of different subgroups on the results. Interaction tests were employed to explore potential heterogeneity between these subgroups. Additionally, GAM, smooth curve fittings, and threshold effect analysis were utilized to investigate the non-linear relationship between SUA and HTG in greater detail.

If the two-sided value *P* < 0.05, the null hypothesis was rejected. All analysis was performed using Empower software (www.empowerstats.com; X&Y solutions, Inc., Boston MA) and R software (version 4.1.2; http://www.R-project.org, R Foundation for Statistical Computing, Vienna, Austria).

## Results

### Basic characteristics of the included participants

**Table 1** presents the weighted baseline characteristics of participants selected from NHANES 2007 to 2018, stratified by the presence of HTG. The analysis included 3,864 participants with HTG. The average age of the HTG group was 50.95 ± 22.04 years, with 58.47% being male and 41.53% female. In comparison, the non-HTG group consisted of 6,163 participants with a mean age of 49.15 ± 30.04 years, with 44.56% being male and 55.44% female. Significant differences between the HTG and non-HTG groups were observed in terms of age, gender, race, education level, PIR, marital status, BMI, drinking, smoking status, PA, hypertension, cholesterol prescription, diabetes, PFC, ALT, AST, SUA, creatinine, blood urea nitrogen, and total cholesterol (all p < 0.05).

**Table 1:** Weighted baseline characteristics of participants.

	Non-Hypertriglyceridemia (n = 6163)	Hypertriglyceridemia (n = 3864)	P-value
**Age (year)**	49.15 ± 30.04	50.95 ± 22.04	<0.001
**Sedentary time (minute)**	394.38 ± 348.66	405.25 ± 297.01	0.0831
**AST (U/L)**	24.39 ± 14.01	26.74 ± 23.15	<0.001
**ALT (U/L)**	23.09 ± 17.62	29.20 ± 27.27	<0.001
**Creatinine (µmol/L)**	77.27 ± 30.84	80.28 ± 28.22	<0.001
**Blood Urea Nitrogen (mmol/L)**	4.98 ± 3.20	5.20 ± 3.17	0.001
**Serum uric acid (µmol/L)**	310.67 ± 112.35	348.06 ± 111.47	<0.001
**Total Cholesterol (mmol/L)**	4.89 ± 1.80	5.45 ± 2.06	<0.001
**Gender (%)**			<0.001
Male	44.56	58.47	
Female	55.44	41.53	
**Race (%)**			<0.001
Mexican American	4.83	7.52	
other Hispanic	4.36	5.17	
Non-Hispanic White	73.27	75.12	
Non-Hispanic Black	11.37	4.93	
Other Race	6.16	7.26	
**Education Level (%)**			<0.001
High school or above high school	92.20	90.44	
Less than high school	7.80	9.56	
**PIR (%)**			0.0099
low-income	12.95	15.15	
middle-income	31.83	32.97	
high-income	55.22	51.88	
**Marital status (%)**			<0.001
**Married**	59.51	63.51	
**living alone**	33.84	5.01	
**living with a partner**	6.65	6.63	
**BMI (kg/m^2^) (%)**			<0.001
underweight or normal	33.89	14.09	
overweight	34.04	33.58	
obese	32.07	52.34	
**Smoking status (%)**			<0.001
Yes	43.17	51.89	
No	56.83	48.11	
**Alcohol intake (%)**			<0.001
Mild	53.94	54.84	
Moderate	35.17	30.57	
Heavy	10.89	14.59	
**Physical activity (%)**			<0.001
Yes	54.42	48.80	
No	45.58	51.20	
**Hypertension (%)**			<0.001
Yes	32.24	44.45	
No	67.76	55.55	
**Cholesterol prescription (%)**			<0.001
Yes	27.05	40.88	
No	72.95	59.12	
**Diabetes (%)**			<0.001
Yes	7.41	15.73	
No	92.59	84.27	

ALT, alanine transaminase; AST, aspartate transaminase; PIR, Ratio of family income to poverty; BMI, body mass index.

All values are presented as proportion (%), or mean ± standard deviation.

### Association between SUA and the HTG

**Table 2** presents the association between SUA and the risk of HTG. A significant positive correlation was observed between SUA and HTG. In Model 1, the odds ratio (OR) was 3.40 (95% CI: 2.84-4.06), indicating a significant association. Model 2, which adjusted for age, gender, race, BMI, education level, PIR, PA, sedentary time, hypertension, diabetes, creatinine, blood urea nitrogen, total cholesterol, ALT, AST, smoking status, drinking, and PFC, also showed a positive association (OR = 3.17, 95% CI: 2.62-3.84). Furthermore, in Model 3, after full adjustment, a positive association between SUA and HTG was still observed (OR = 1.77, 95% CI: 1.44-2.18).

**Table 2:** Weighted Multivariate logistic regression models of SUA with hypertriglyceridemia.

log-SUA (umol/L)	OR^a^(95% CI), P-value		
	Model 1^b^	Model 2^c^	Model 3^d^
**Continuous**	3.40 (2.84, 4.06) <0.001	3.17 (2.62, 3.84) <0.001	1.77 (1.44, 2.18) <0.001
**Categories**			
**Quartile 1 (≤8.06)**	**Reference**	**Reference**	**Reference**
**Quartile 2 (8.06-8.33)**	1.48 (1.22,1.80) <0.001	1.41 (1.15,1.73) <0.001	1.17 (0.95,1.45) 0.14
**Quartile 3 (8.34-8.57)**	2.20 (1.81,2.70) <0.001	2.01 (1.63,2.48) <0.001	1.43 (1.16,1.78) 0.0018
**Quartile 4 (>8.57)**	3.29 (2.73,3.97) <0.001	3.02 (2.46,3.69) <0.001	1.68 (1.36,2.08) <0.001

SUA, serum uric acid; 95% CI, 95% confidence interval; OR, odds ratio.

In sensitivity analysis, SUA is converted from a continuous variable to a categorical variable (quartile); OR^a^, effect size; Model 1^b^, no covariates were adjusted; Model 2^c^, adjusted for gender, age, and race; Model 3^d^, adjusted for gender, age, race, PA, sedentary time, ALT, AST, creatinine, blood urea nitrogen, education level, the ratio of family income to poverty, marital status, body mass index, alcohol intake, smoking status, hypertension, cholesterol prescription, total cholesterol, and diabetes.

To gain further insights into the relationship between SUA and HTG, log-transformed SUA was categorized into quartiles. In the fully adjusted Model 3, when comparing the highest quartile (Q4) with the lowest quartile (Q1), the OR was 1.68 (95% CI: 1.36-2.08), indicating a stable positive association between higher SUA levels and HTG.

### Subgroup analysis

While informative, subgroup analysis was conducted to further assess the robustness of the association between SUA and HTG. Interaction tests were also performed to assess the influence of different variables (**Supplementary material 2**). The results of the subgroup analysis revealed a consistent positive correlation between SUA and HTG across different subgroups, indicating the robustness of the association. Notably, no significant interactions were observed for gender, BMI, smoking status, drinking, hypertension, diabetes, and PA, suggesting that the association was not dependent on these variables (all *p* for interaction > 0.05).

However, age was found to significantly impact the strength of the SUA-HTG association (all *p* for interaction < 0.05). The results indicated that participants under the age of 60 were at a higher risk compared to those aged 60 and older, with an odds ratio of 2.47 (95% CI: 1.96-3.12). This suggests that age plays a role in modifying the association between SUA and HTG, with younger individuals exhibiting a stronger association.

### Identification of non-linear relationship

**Figure 2:**
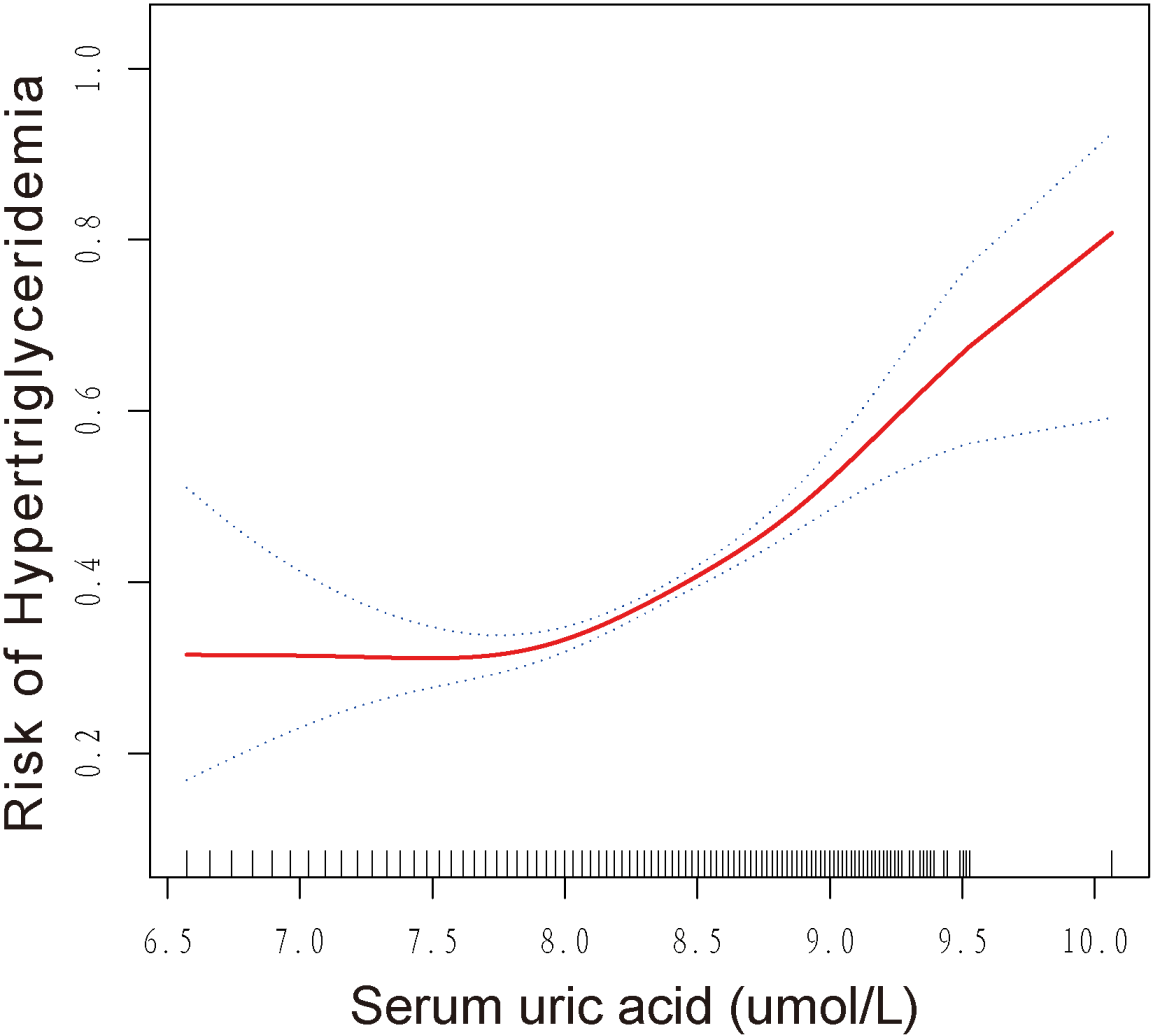
smoothed curve fitting: Dose-response relationship between SUA and hypertriglyceridemia.

This study revealed a non-linear relationship between SUA and HTG, as demonstrated by the results of GAM and smoothed curve fitting presented in **Figure 2**. The log-likelihood ratio test showed a *p*-value of less than 0.001 when comparing the linear regression model to a two-piecewise linear regression model, indicating that the two-piecewise linear regression model provided a better fit for the data. Using the two-piecewise linear regression model and recursive algorithm, **Table 3** presents the findings. The point of inflection in the U-shaped association between SUA and HTG was identified as 7.86 umol/L for log2-SUA. To the left of the inflection point, the effect size (log2 transformed) was 0.76 (95% CI: 0.45, 1.28) with a *p*-value of 0.30, suggesting a non-significant association. However, to the right of the inflection point, SUA showed a significant positive correlation with HTG. The effect size (log2 transformed) was 2.16 (95% CI: 1.82, 2.56) with a *p*-value of <0.001, indicating a strong and significant association between higher SUA levels and HTG.

**Table 3:** Threshold effect analysis of SUA on HTG using two-piecewise linear regression model.

log2-SUA (umol/L)	Adjust OR (95% CI) P value
Fitting by linear regression model	1.84 (1.60, 2.13) <0.0001
Fitting by two-piecewise linear regression model	
Inflection point	7.86
< 7.86	0.76 (0.45, 1.28) 0.30
> 7.86	2.16 (1.82, 2.56) <0.0001
Log likelihood ratio test	<0.001

Adjusted for gender, age, race, PA, sedentary time, ALT, AST, creatinine, blood urea nitrogen, education level, ratio of family income to poverty, marital status, body mass index, alcohol intake, smoking status, hypertension, cholesterol prescription, total cholesterol,and diabetes.

## Discussion

Our study revealed a significant positive association between SUA and HTG. Subgroup analysis indicated that this association was consistent across different subgroups. Interaction tests demonstrated that the association was independent of gender, BMI, smoking status, alcohol intake, physical activity, hypertension, and diabetes. Interestingly, participants under the age of 60 had a higher risk of developing HTG compared to those aged 60 and older. Moreover, we observed a U-shaped association between SUA and HTG, with an inflection point identified at 7.86 umol/L.

The association between SUA and Metabolic Syndrome (MetS) has been extensively studied in previous research. MetS is characterized by the abnormal accumulation of multiple metabolic components, such as obesity, HTG, low high-density lipoprotein cholesterol (HDL-C), hypertension, and insulin resistance (IR). Several studies have indicated that SUA might serve as an independent risk factor for MetS (42, 43). These studies have provided evidence supporting the potential role of SUA in the development of MetS (44–46). The result of a cohort study revealed that high SUA concentrations may increase the risk of MetS among Chinese adults (47). There is evidence that SUA may be associated with IR, which is one of the diagnostic indicators of MetS (48). Furthermore, Xanthine oxidoreductase (XO), an important enzyme involved in the production of SUA, has been suggested to play a crucial role in the development of MetS (49). Animal experiments have shown that lowering SUA levels can prevent and reverse MetS features in fructose-fed rats, including lower blood pressure, reduced serum triglycerides, decreased hyperinsulinemia, and weight gain (50). These findings, along with our study results, support the connection between SUA and the development of MetS.

The underlying mechanism linking SUA and HTG remains unclear. However, studies have suggested that high intracellular SUA levels can lead to increased oxidative stress in mitochondria. In an *in vitro* study by Yang et al. (51), hepatocytes treated with different concentrations of SUA exhibited increased apoptotic activity, accumulation of Reactive Oxygen Species (ROS), and elevated 8-hydroxydeoxyguanosine levels compared to control cells, indicating mitochondrial DNA damage. This mitochondrial dysfunction could contribute to the release of citrate into the cytosol, initiating lipogenesis and triglyceride synthesis (52). In addition, Wang et al. (53) found that the prevalence of the E2 allele of ApoE was correlated with increased SUA level. ApoE is known to play a role in regulating lipoprotein metabolism. Studies have suggested that ApoE may contribute to decreased clearance of very low-density lipoprotein (VLDL) through the interaction with the hepatic remnant receptor, leading to elevated levels of VLDL cholesterol and VLDL triglycerides (54). In addition, it has been suggested that elevated SUA may be associated with reduced lipase activity (30). In the study by Zheng et al. (31), it was found that elevated SUA levels may hinder the breakdown of triglycerides (TG) by reducing lipase activity. This inhibition of TG catabolism could contribute to a higher prevalence of HTG in individuals with high SUA levels.

In our subgroup analysis, we found that the association between SUA and HTG was more pronounced in participants under 60 years old compared to those over 60 years old. This observation is consistent with previous evidence suggesting that the health effects of SUA are stronger in younger individuals (55). For example, Kawamoto et al. (56) reported higher risk factor values for cardiovascular disease induced by SUA in women aged <55 compared to older participants. However, the underlying mechanism for this age-related effect of SUA on HTG is not yet well understood and requires further exploration in future scientific research. Moreover, our threshold effects analysis revealed that the inflection point for the association between log2-SUA and HTG was 7.86 umol/L. Beyond this threshold, there was a significantly higher risk of HTG. However, more experimental studies are needed to determine more precise threshold effects and to elucidate the mechanisms involved.

This study has several strengths that contribute to its scientific value. Firstly, it utilized a large sample size of 10,027 participants, providing robust statistical power for accurately assessing the association between SUA and HTG. Secondly, the study utilized data from the NHANES database, which is a nationally representative population-based sample. By incorporating appropriate sampling weights, the study results can be generalized to the entire US population. Lastly, the study accounted for various confounding covariates based on previous research and clinical experience, minimizing the potential bias caused by these factors.

However, there are certain limitations that should be acknowledged. Firstly, being a cross-sectional study, it is unable to establish a causal relationship between SUA and HTG. Further longitudinal studies are needed to explore the temporal nature of this association. Secondly, despite adjusting for several potential confounding factors, it is possible that other unmeasured variables might have influenced the results. Additionally, caution should be exercised when extrapolating the findings to populations outside of the United States, as the study was restricted to American participants. Finally, due to the limited amount of relevant research published in the last five years, not all of our primary references are recent works, which may have an impact on the timeliness of this study.

## Conclusions

In conclusion, our study demonstrates a strong positive association between SUA levels and HTG in the US adult population, indicating that elevated SUA may contribute to an increased risk of HTG. These findings highlight the importance of managing and controlling SUA levels in HTG patients to prevent disease progression. Crucial for future research is to conduct large-scale and high-quality prospective studies to validate our conclusions and further explore the underlying mechanisms of this association.

## Data availability statement

The datasets presented in this study can be found in online repositories. The names of the repository/repositories and accession number(s) can be found below: https://www.cdc.gov/nchs/nhanes/.

## Ethics statement

The studies involving human participants were reviewed and approved by National Center for Health Statistics (NCHS). The patients/participants provided their written informed consent to participate in this study.

## Author contributions

Conception and design of the study: M-YT, QZ. Data collection and analysis: M-YT, C-YM, FL, QZ. Draft paper: M-YT, C-YM, QZ. Approve the final paper to be published: All authors.
